# Randomized controlled trial evaluating synbiotic supplementation as an adjuvant therapy in the treatment of Parkinson’s disease

**DOI:** 10.1007/s10787-025-01752-8

**Published:** 2025-05-28

**Authors:** Mohamed E. Ramadan, Tarek M. Mostafa, Azza A. Ghali, Dalia R. El-Afify

**Affiliations:** 1 Clinical Pharmacy Department, Faculty of Pharmacy, Tanta University, Tanta, 31111 Egypt; 2 Neuropsychiatry Department, Faculty of Medicine, Tanta University, Tanta, Egypt

**Keywords:** Synbiotic, Parkinson’s disease, MDS-UPDRS, BDNF, TNF-α, MDA

## Abstract

**Background and aim:**

Neuroinflammatory mechanisms have been closely related to the microbiota-gut-brain axis and could lead to degeneration of dopaminergic neurons with subsequent development and progression of Parkinson’s disease (PD). Targeting this pathway for the treatment of PD has sparked a lot of interest. Hence, this study investigates the therapeutic potential of a synbiotic supplement, in conjunction with l-dopa for the management of PD.

**Methods:**

This randomized controlled trial enrolled 66 Parkinson's disease patients, who were randomly assigned to two groups: a control group (*n* = 33) receiving standard l-dopa/carbidopa (100/25 mg) therapy three times daily for three months, and a synbiotic group (*n* = 33) receiving the same l-dopa/carbidopa regimen with two sachets of the synbiotic supplement daily for three months. The outcome measures included assessment of Movement Disorder Society Unified Parkinson's Disease Rating Scale (MDS-UPDRS) and serum levels of tumor necrosis factor-alpha (TNF-α), malondialdehyde (MDA), brain-derived neurotrophic factor (BDNF), and α-synuclein (α-Syn). Blood samples were collected from all patients for biomarker analysis in serum.

**Results:**

Three months after intervention, the synbiotic group demonstrated significantly greater improvement in motor and non-motor symptoms compared to the control group evidenced by the change in the scores of each part of the MDS-UPDRS. Concurrently, the synbiotic group exhibited significantly lower serum levels of pro-inflammatory marker TNF-α and oxidative stress marker MDA, and significantly higher levels of the neuroprotective factor BDNF.

**Conclusion:**

Supplementing with synbiotics exhibits promising neuroprotective and therapeutic effects in the treatment of Parkinson’s disease patients.

**Clinicaltrials.gov registration number:**

NCT05576818. Retrospectively registered in October 2022.

## Introduction

Parkinson's disease (PD) is a chronic, progressive neurodegenerative disorder that affects multiple systems, caused by the degeneration of dopaminergic neurons in the substantia nigra. The disease is characterized by motor symptoms, including rigidity, rest tremors, bradykinesia, and the loss of postural reflexes, as well as non-motor symptoms such as gastrointestinal, urogenital, sensory, neuropsychiatric, and olfactory disturbances (Radad et al. 2023). According to the World Health Organization (WHO) in 2019, PD was responsible for 5.8 million disability-adjusted life years (DALYs), marking an 81% increase since 2000. It also led to approximately 329,000 deaths, more than double the number from 2000, making PD the fastest-growing brain disorder worldwide (Xu et al. 2024).

The pathogenesis of PD remains elusive. Although, the role of neuro-inflammation in the etiology of PD was previously discussed in the literature. Parkinson’s disease has been linked to persistent activation of microglia by the pro-inflammatory molecules, or microgliosis, which then express inflammatory molecules such as pro-inflammatory mediators and reactive species (Jayaram and Krishnamurthy, 2021). Furthermore, many studies reported elevated levels of pro-inflammatory cytokines in patient with PD (Liu et al. 2022; de Araújo et al. 2022), and the existence of a positive relationship between inflammation and the severity of PD symptoms (Scalzo et al. 2010b; Lindqvist et al. 2012; Diaz et al. 2022). Tumor necrosis factor-alpha (TNF-α) in particular was reported to be elevated in patients with PD as compared to healthy controls (El-Kattan et al. 2022; Xiromerisiou et al. 2022; Fu et al. 2023). The progression of PD was reported to be significantly influenced by oxidative stress and lipid peroxidation-related free radicals as well, which cause dopaminergic neurons malfunction and degeneration, and subsequently, the development of PD (Olufunmilayo et al. 2023). Malondialdehyde (MDA) is the end product of lipid peroxidation, and it can be assessed as a marker for oxidative stress in PD patients, since its level was reported to be elevated in patients with PD as compared to healthy controls (Naduthota et al. 2017). It was also previously reported that patients in higher Hoehn and Yahr stages of PD (stages 3 and 4) had higher MDA levels than patients in lower stages (stages 1 and 2), which reflects the correlation between MDA and the severity of PD (Fedorova et al. 2019). On the other hand, Brain derived neurotrophic factor (BDNF) is a neurotrophin which can prevent neuronal death. It was postulated that, a decline in neurotrophins expression, particularly BDNF, is common with aging and neurodegenerative illnesses, and could represent a factor in the degeneration and death of neurons (Palasz et al. 2020). Previous studies reported lower levels of BDNF in patients with PD as compared to healthy controls (Wang et al. 2017; Huang et al. 2021; Badr et al. 2023). Furthermore, it has been demonstrated that, α-synuclein (α-Syn) suppresses vesicular priming; decreases synaptic contact, and keeps tyrosine hydroxylase, an enzyme essential for the generation of dopamine, inactive. Thus, it was hypothesized that an increase in α-Syn levels could decrease dopamine neurotransmission (Cook et al. 2012; Butler et al. 2017). In previous studies, α-Syn was reported to be elevated in the serum of patients with PD as compared to healthy controls, with presence of a positive correlation between α-Syn levels and the severity of PD symptom (Bougea et al. 2019; Chang et al. 2020; Wang et al. 2019).

The relationship between the microbiota-gut-brain axis and the pathogenesis of PD is a more recent discovery, where numerous studies reported its contribution to the aforementioned pathogenic mechanisms. It was also reported that the bioavailability of l-dopa, the breakthrough drug used for treatment of PD, could be affected by the gut microbiota. Hence, the involvement of the microbiota-gut-brain axis in PD pathology could be used as a modifiable target for the treatment of PD (Roy Sarkar and Banerjee 2019; van Kessel et al. 2020; Tan et al. 2021a; Wang et al. 2021; Chen and Mor 2023).

One of the treatment options to correct intestinal microbiota are probiotics; which are live microorganisms that have beneficial effects (Uyar and Yildiran 2019). Previous animal and human studies revealed an improvement in PD symptoms after being treated with different probiotics, with Lactobacillus acidophilus being among the strains demonstrating the highest activity in reducing markers of inflammation in PD patients on a cellular level (Magistrelli et al. 2019; Tamtaji et al. 2019; Mirzaei et al. 2022).

Prebiotics, on the other hand, are dietary components which can promote the growth of beneficial bacteria in the gut over the harmful ones (Uyar and Yildiran 2019). Administration of prebiotics also demonstrated beneficial effects in improving motor function and reducing α-Syn aggregation and neuroinflammation in α-Syn overexpressing mice (Abdel-Haq et al. 2022).

Synbiotics are a mixture of probiotics and prebiotics which exhibit both of their characteristics. Synbiotics were introduced for overcoming possible problems regarding probiotics’ viability in the gastrointestinal tract. Thus, a suitable blend of probiotics and prebiotics ought to warrant a better effect, compared to the efficacy of each component alone (Markowiak and Śliżewska 2017; Zhang et al. 2024).

Hence, based on the aforementioned data, we aimed to evaluate the safety and efficacy of a synbiotic preparation containing Lactobacillus acidophilus as a probiotic with inulin as a prebiotic as an adjuvant therapy with l-dopa in the treatment of PD.

## Patients and methods

### Study design and patient population

This study was a randomized, parallel, controlled trial that enrolled 66 newly diagnosed PD patients recruited from the Neuropsychiatry Department at Tanta University Hospital between August 2022 and April 2024. Diagnosis was performed according to the UK Parkinson's Disease Society Brain Bank Diagnostic Criteria (Hughes et al. 1992). Patients were randomly assigned to two groups: a control group (*n* = 33) receiving standard l-dopa/carbidopa (100/25 mg) therapy three times daily for three months, and a synbiotic group (*n* = 33) receiving the same l-dopa/carbidopa regimen (100/25 mg therapy three times daily) plus two sachets daily of the synbiotic preparation for three months. Allocation concealment was performed by sealed opaque envelopes with sequential numbers. After signing the consent to participate in the study, the sealed opaque envelope was opened and the patient was enrolled into the respective group.

### Study medications

The tested synbiotic supplement used in our study was Livia® which was manufactured by Pharma Zad for Pharmaceutical Industries and was available for purchase through Diamond Labs, Egypt. It is formulated as a powdered preparation packed in aluminum sachets, intended for dissolution in 120 ml water to be administered orally. Each sachet contains 10 billion colony forming units of Lactobacillus acidophilus probiotic and 3 g of inulin prebiotic as the active ingredients, with excipients including colloidal silicon dioxide, steviol glycoside powder and natural strawberry flavor. The standard PD medication used in our study was Levocar® which was manufactured by and purchased through Alphacure Pharmaceuticals, Egypt. It is formulated as controlled release tablets for oral intake. Each tablet contains 100 mg of levodopa as the active ingredient with 25 mg of carbidopa. The study medications were given to the patients by registered neurologists at the Neuropsychiatry Department, Tanta University Hospital.

### Ethical approval

The Research Ethics Committee of Tanta University granted ethical approval for this study (approval code: 35539/6/22). This study adhered to the ethical principles of the 1975 Declaration of Helsinki. All participants provided a written informed consent. The study is registered on Clinicaltrials.gov with the trial registration number NCT05576818.

### Inclusion criteria

This study included individuals newly diagnosed with PD who had not previously received any treatment. Participants were between 45 and 65 years old, of both sexes, and classified in stages 1–4 on the Hoehn and Yahr scale (stage 1: Unilateral involvement only; stage 2: Bilateral involvement without impairment of balance; stage 3: Mild to moderate involvement; some postural instability but physical independence, needs assistance to recover from pull test; stage 4: Severe disability, still able to walk or stand without assistance)

### Exclusion criteria

The exclusion criteria included smokers; patients who are currently using or used antibiotics in the preceding month; patients who currently using or used other probiotic containing preparations in the preceding two weeks; patients on anti-oxidant and/or anti-inflammatory medications; patients who were scheduled to undergo or already underwent GIT surgery; and those who received artificial enteral or intravenous nutrition; as well as patients with known allergies to probiotics; patients with depression, psychosis, thyroid disorders, inflammatory conditions, and conditions involving oxidative stress. Patients in stage 5 of the Hoehn and Yahr scale were also excluded (Stage 5: wheelchair bound or bedridden unless aided).

### Methods

#### Demography, anthropometric data and physical examination

All patients were subjected to physical examination, demographic data collection (age, sex, and medication history), and weight and height measurements.

#### Clinical assessment

The Hoehn and Yahr staging scale was applied on all patients upon admission to determine their eligibility for the study according to the study’s inclusion and exclusion criteria (Goetz et al. 2004).

#### Blood samples collection

5 ml venous blood samples were collected in plain tubes via sterile venipuncture from each participant at baseline and after three months of intervention using disposable syringes, following minimal venous stasis and without frothing. Blood samples were allowed to clot and centrifuged shortly after collection at 3000 rpm. Sera were extracted from the collected blood samples and kept frozen at – 80 °C for subsequent analysis. Serum levels of tumor necrosis factor-alpha (TNF-α), malondialdehyde (MDA), brain-derived neurotrophic factor (BDNF), and α-synuclein (α-Syn) were assayed within 6 months after storage at maximum using commercially available enzyme-linked immunosorbent assay (ELISA) kits. The ELISA assays were conducted according to the manufacturer's instructions which implicated a double-antibody sandwich technique (SunRed Biological Technology Shanghai, China: Catalogue numbers 201-12-0083, 201-12-1372, 201-12-1303, 201-12-1314, respectively).

#### Assessment of participants' adherence and drug tolerability

Weekly phone calls and monthly in-person meetings were used to check on patients' compliance and report any medication related adverse reactions using adverse drug reactions reporting form. Patients’ compliance with the study medication was evaluated by counting the number of tablets and empty sachets as well as the rate at which the prescription was refilled. Patients who did not adhere to their medication regimen or who altered their therapy during the trial were excluded from the study.

### Primary and secondary outcome of the study

#### Primary outcome

The MDS-UPDRS (Movement Disorder Society-Unified Parkinson's Disease Rating Scale) is a comprehensive clinical tool for assessing the severity of PD symptoms. It consists of four parts, of which Parts I, II, and III were utilized in this study. Part I assesses non-motor aspects of daily living, such as cognitive impairment, hallucination and psychosis, mood, features of dopamine dysregulation syndrome, sleep disturbances, constipation, urinary problems, pain, lightheadedness, and fatigue. Part II evaluates motor aspects of daily living (i.e., the impact of motor symptoms on daily activities), including speech, saliva and drooling, performing tasks such as eating, chewing, dressing, turning in bed, getting out of bed, walking and balance, doing hobbies and other activities, handwriting and tremors during activities. Part III focuses on a clinician-rated motor examination, assessing symptoms such as rigidity, bradykinesia, tremors and postural stability by physical examination as well as by asking the patient to perform activities such as finger tapping, toe tapping, hand and leg movement, standing up and walking. Each item is scored on a scale of 0 to 4, with higher scores indicating greater severity. The primary outcome in our study was the improvement from baseline scores of each part individually. The sum score for each individual part was reported as recommended by the MDS-UPDRS task force (Goetz et al. 2008).

The scale was applied to all patients by registered neurologists at Neuropsychiatry Department, Tanta University Hospital. The scale was assessed 2–3 hours after the first dose of l-dopa/carbidopa (after running of the study) and again 2–3 hours after the last dose of l-dopa/carbidopa (at the end of the study) to evaluate the changes in the severity of symptoms and symptoms progression during the “on” state (i.e., the patient is under the influence of l-dopa/carbidopa treatment).

#### Secondary outcome

Inflammatory marker TNF-α, oxidative stress marker MDA, neurotrophic neurotrophin BDNF, and α-Syn protein were assessed in the serum samples collected from all patients at baseline and after 3 months. The secondary outcome was the change in serum levels of the measured biomarkers.

### Sample size calculation

An estimated sample size of 30 patients in each group seems adequate to detect a mean change of -5 in MDS-UPDRS with α error = 0.05, β error = 0.2, and a statistical power of 80%, based on a previous study (Tamtaji et al. 2019). Assuming that the attrition rate is 10%, the sample size was 33 patients in each group.

### Statistical analysis

Statistical analysis was performed using the Statistical Package for Social Science (SPSS version 26, IBM Corporation Software Group, USA). Frequencies and percentages were used to express nominal variables. Means, standard deviations, and ranges were used to express parametric numerical variables, while non-parametric numerical variables were expressed as medians and interquartile ranges. Data normality was assessed by the Shapiro–Wilk test. Paired and unpaired t-tests were employed to compare mean values within the same group and between the two groups before and after treatment for parametric data. For non-parametric data, the Wilcoxon signed-rank test and Mann–Whitney U test were used to compare median values within the same group and between the two groups before and after treatment. Categorical data were analyzed by the chi-square test, and Fisher's exact test was used for analyzing any reported adverse effects. Correlation between variables were examined using Pearson correlation test. Statistical significance was set at a *p*-value < 0.05

## Results

**Fig. 1:**
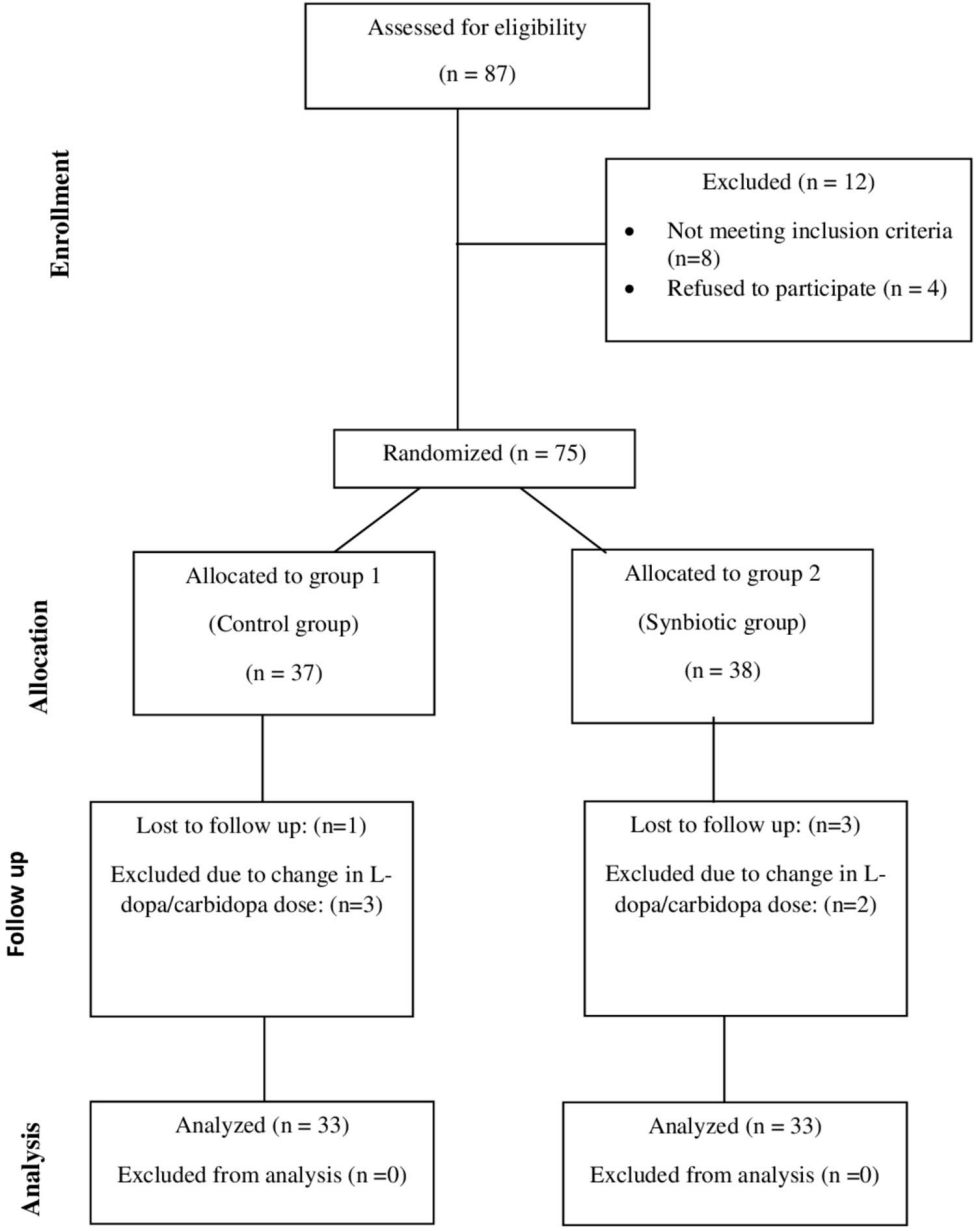
CONSORT Participants flowchart

Figure 1 illustrates the participant flow diagram according to the CONSORT guidelines. Of the 87 patients screened for eligibility, 12 were excluded: 8 were deemed ineligible for participation, and 4 declined participation. A total of 75 patients were then randomly enlisted into two groups: the control group (*n* = 37) receiving l-dopa/carbidopa and the synbiotic group (*n* = 38) receiving l-dopa/carbidopa plus the synbiotic supplement. During the follow-up period, 9 patients were lost to follow-up: 1 in the control group and 3 in the synbiotic group due to loss of contact, and 3 in the control group and 2 in the synbiotic group due to the necessity of altering their l-dopa/carbidopa dose. A total of 33 patients per group were included in the final analysis.

Since our study protocol mandates a constant l-dopa/carbidopa dose for all participants throughout the study duration to avoid interference with the outcome related to the implication of the synbiotic supplementation to get a clear and non-biased clinical and biochemical response to this supplement which is still under investigation, an intention to treat analysis was not employed in the analysis and instead per protocol analysis was implicated.

### Demographic data, anthropometric measurements and Hoehn and Yahr staging scale of the studied patients

No significant between-group differences were observed in terms of age, weight, height, gender, and Hoehn and Yahr stages at baseline, as shown in Table 1.

**Table 1:** Demographic data, anthropometric measurements and Hoehn and Yahr staging for the two study groups

Variable		Control group		Synbiotic group		*P*-value
		Mean ± S.D	Range	Mean±S.D	Range	
Age (years)		55.7±5.91	47–65	56.06±6.2	45–65	0.810
Weight (kg)		89.97±10.51	75–114	90.61±10.92	75–117	0.809
Height (cm)		170.61±9.61	154–186	169.97±9.83	153–183	0.791
*Gender *		*N(%) *		*N(%) *		
Male		16(48.5%)		15(45.5%)		0.807
Female		17(51.5%)		18(54.5%)		
Total		33(100%)		33(100%)		
*H and Y staging scale*		*N(%) *		*N(%) *		
Stage 1		8 (24.2%)		7 (21.2%)		0.997
Stage 2		10 (30.3%)		10 (30.3%)		
Stage 3		10 (30.3%)		11 (33.3%)		
Stage 4		5 (15.2%)		5 (15.2%)		
Stage 5		0 (0%)		0 (0%)		
	Median (Range)	2 (1-4)		2 (1–4)		
	Total	33 (100%)		33 (100%)		

*kg* kilogram, *cm* centimeter, *H and Y* Hoehn and Yahr, *stage 1* unilateral involvement only, *stage 2* bilateral involvement without impairment of balance, *stage 3* mild to moderate involvement, some postural instability but physical independence, needs assistance to recover from pull test, *stage 4* severe disability, still able to walk or stand without assistance and *stage 5* wheelchair bound or bedridden unless aided

Numerical data are presented as mean ± standard deviation and analyzed using unpaired t-test

Categorical data are presented as number and percent and analyzed using chi-square test

### The change in MDS-UPDRS Parts I, II and III

Baseline MDS-UPDRS scores were not significantly different between both groups. Three months after intervention, the synbiotic group exhibited a significantly lower score in Part I compared to the control group (*p* = 0.016), while no significant between-group differences were observed in Parts II and III. However, the synbiotic group demonstrated greater improvements from baseline across all three MDS-UPDRS parts compared to the control group (p<0.001 for Parts I and II, and p=0.004 for Part III). Compared to baseline, the control group showed a significant improvement only in Part II (*p* = 0.032), while the synbiotic group exhibited significant improvements in all three parts (*p* < 0.001 for all), as detailed in Table 2.

**Table 2:** Changes in MDS-UPDRS Parts I, II and III for the two study groups

Variable	Control group		Synbiotic group		*P*_1_-value
	Mean ±S.D	Range	Mean ±S.D	Range	
*Part I*					
Before treatment	20.85±8.71	8–36	20.91±8.15	7–35	0.971
After treatment	20.33±8.29	8–36	15.76±6.66	6–29	0.016*
Mean change (SD)	-0.52 (2.12)		-5.15 (2.88)		<0.001*
*P*_2_-value	0.173		<0.001*		–
*Part II*					
Before treatment	19.88±7.78	9–32	20.21±8.09	9–32	0.865
After treatment	18.94±7.22	8–33	17±7.29	6–29	0.282
Mean change (SD)	-0.94 (2.41)		-3.21 (2.62)		<0.001*
*P*_2_-value	0.032*		<0.001*		–
*Part III*					
Before treatment	30.61±15.58	14–59	32.27±19.13	7–65	0.699
After treatment	29.82±15.53	3–61	27.85±16.75	4–55	0.622
Mean change (SD)	-0.58 (5.32)		-4.15 (4.24)		0.004*
*P*_2_-value	0.425		<0.001*		–

*MDS-UPDRS* Movement disorder society unified Parkinson’s disease rating scale, *Part I* non-motor aspects of experiences of daily living, *Part II* motor aspects of experiences of daily living, *Part III* motor examination, *p1-value* difference between both groups both before and after treatment, *p2-value* difference within the same group before versus after treatment

Data are presented as mean ± standard deviation and analyzed using unpaired t-test to compare between the two groups, and paired t-test to compare between before and after treatment within the same group

*Significant difference (*p* <0.05)

### The change in serum levels of measured biological markers

**Fig. 2:**
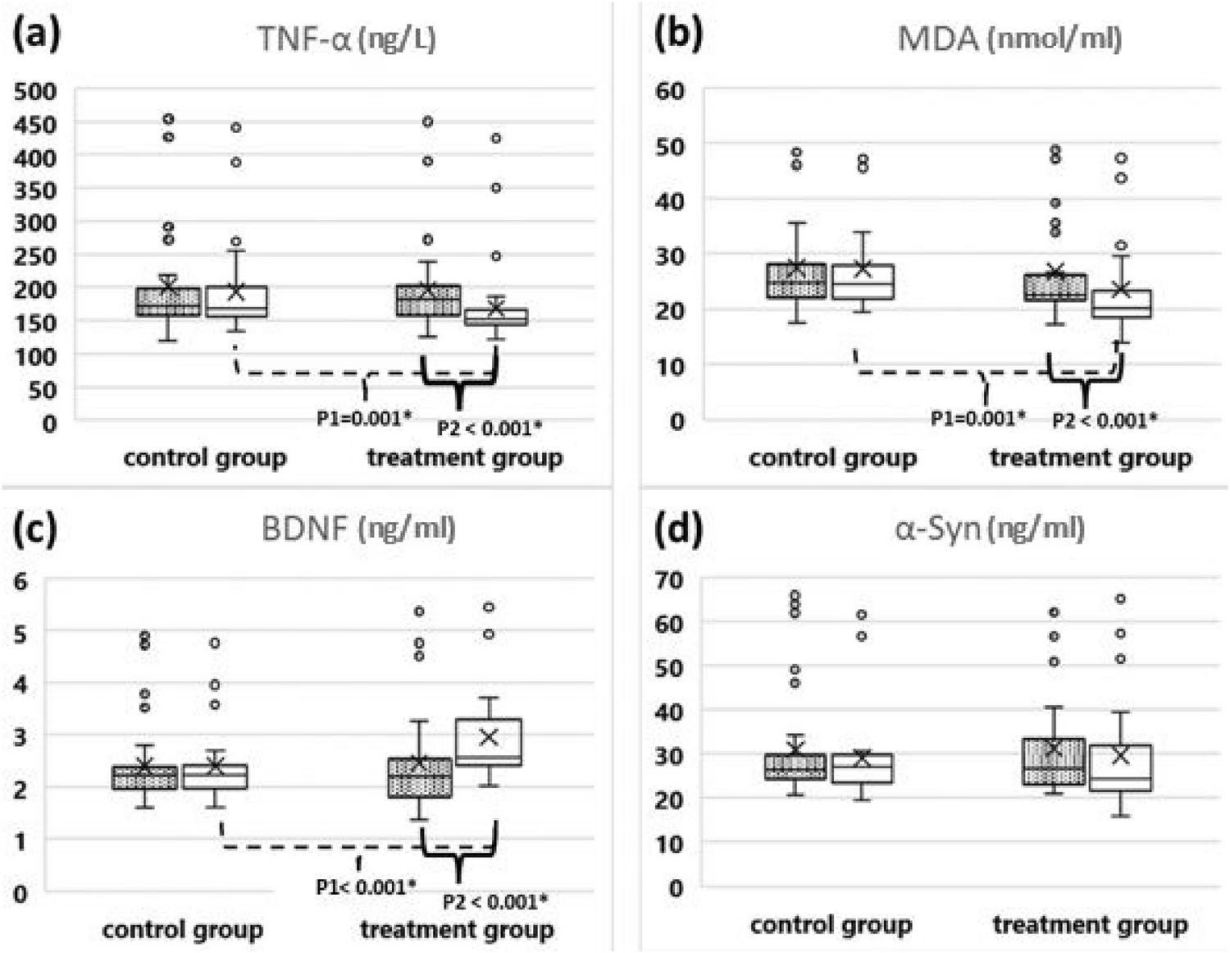
**a** The change in serum level of TNF-α throughout the treatment course **b** The change in serum level of MDA throughout the treatment course **c** The change in serum level of BDNF throughout the treatment course **d** The change in serum level of α-Syn throughout the treatment course. TNF-α: tumor necrosis factor-alpha, MDA: malondialdehyde, BDNF: brain derived neurotrophic factor, α-Syn: α-synuclein protein. P1: difference between both groups after treatment. P2: difference between before and after treatment within the same group. Data are presented as median and interquartile range and analyzed using Mann Whitney U test to compare between the two groups, and Wilcoxon signed rank test to compare between before and after intervention within the same group. The dotted boxes represent data before treatment. The plain boxes represent data after treatment. The dashed brackets point to the difference between both groups after treatment. The solid brackets point to the difference between before and after treatment within the same group. * Statistical significance (*p* < 0.05)

No significant baseline differences were observed between groups in serum levels of TNF-α, MDA, BDNF, and α-Syn (*p* > 0.05 for all). After three months, the synbiotic group exhibited significantly lower serum levels of TNF-α and MDA (*p* = 0.001 for both) and significantly higher levels of BDNF (*p* < 0.001) compared to the control group, while no significant between-group differences were observed in α-Syn levels. Compared to baseline, the control group showed no significant differences in any of the measured biomarkers. In contrast, the synbiotic group demonstrated significantly lower TNF-α and MDA levels (*p* < 0.001 for both) and a significantly higher BDNF level (*p* < 0.001), while a non-significant difference in α-Syn levels was observed in the synbiotic group (*p* = 0.148), as illustrated in Fig. 2.

### The correlation between the measured biological parameters and the different parts of MDS-UPDRS

The correlation analysis between the measured biological markers after treatment revealed that TNF-α showed significant positive correlation with Parts I and III in both groups and with Part II in the synbiotic group only, MDA exhibited significant positive correlations with Parts I, II, and III in the synbiotic group, BDNF showed a significant negative correlation with Part I and Part III in both groups and with Part II in the synbiotic group only, and lastly, α-Syn demonstrated a significant positive correlation with Part I and Part III in both groups and with Part II in the synbiotic group only, while it also showed a significant negative correlation with Part II in the control group. These results are postulated in Table 3.

**Table 3:** The correlation between the measured biological parameters and the different parts of MDS-UPDRS after the treatment course

Markers		TNF-α	MDA	BDNF	α-Syn
*Part I*					
Control group	*R*_1_ (*P*_1_-value)	0.841 (<0.001*)	0.152 (0.400)	− 0.718 (<0.001*)	0.823 (<0.001*)
Synbiotic group	*R*_2_ (*P*_2_-value)	0.789 (<0.001*)	0.842 (<0.001*)	− 0.821 (<0.001*)	0.910 (<0.001*)
*Part II*					
Control group	*R*_1_ (*P*_1_-value)	− 0.335 (0.056)	− 0.123 (0.496)	0.270 (0.128)	− 0.371 (0.034*)
Synbiotic group	*R*_2_ (*P*_2_-value)	0.802 (<0.001*)	0.876 (<0.001*)	− 0.817 (<0.001*)	0.944 (<0.001*)
*Part III*					
Control group	*R*_1_ (*P*_1_-value)	0.522 (0.002*)	− 0.026 (0.888)	− 0.560 (0.001*)	0.501 (0.003*)
Synbiotic group	*R*_2_ (*P*_2_-value)	0.788 (<0.001*)	0.875 (<0.001*)	− 0.795 (<0.001*)	0.926 (<0.001*)

*MD-UPDRS* Movement disorder society unified Parkinson’s disease rating scale, *Part I* non-motor aspects of experiences of daily living, *Part II* motor aspects of experiences of daily living, *Part III* motor examination, *TNF-α* tumor necrosis factor-alpha, *MDA* malondialdehyde, *BDNF* brain derived neurotrophic factor, *α-Syn* α-synuclein protein, *R1* correlation coefficient for the control group, *R2* correlation coefficient for the Synbiotic group, *P1-value* probability of significance in the control group, *P2-value* probability of significance in the synbiotic group

Correlation analysis was done using Pearson correlation test

*Statistical significance (*p* <0.05)

### Safety and tolerability of the study medications

Regarding drug-related complications; no adverse reactions were observed or reported by the patients in both groups.

## Discussion

In our study, to evaluate the clinical outcome regarding motor and non-motor symptoms at baseline and 3 months after intervention, we used the scores of Parts I, II, and III of MDS-UPDRS through assessing the score of each part individually while patients with PD were in an “on” state (2–3 h after receiving their first and their latest l-dopa dose). Our results showed that, after 3 months of treatment, Part I showed a significantly lower mean score in the synbiotic group as compared to the control group, which is in agreement with several previous clinical studies that reported that probiotic and synbiotic supplementation significantly improved constipation and some other non-motor symptoms in PD patients (Barichella et al. 2016; Ibrahim et al. 2020; Tan et al. 2021b; Mehrabani et al. 2023; Ghalandari et al. 2023; Andreozzi et al. 2024). While no significant between-group differences were observed in MDS-UPDRS Parts II and III after 3 months, which is consistent with previous findings (Ibrahim et al. 2020; Mehrabani et al. 2023; Ghalandari et al. 2023). Despite that, our results revealed that the synbiotic group demonstrated significantly greater improvement from baseline in all three MDS-UPDRS parts compared to the control group. which is in accordance with Tamtaji et al. (2019) who demonstrated that supplementation with a blend of probiotic strains caused significant decrease in the overall score of the MDS‐UPDRS in patients with PD with a significantly higher mean change from baseline score compared to placebo (Tamtaji et al. 2019). However, and in contrast to our data, Tamtaji et al. (2019) did not clarify the score of each part of the MDS-UPDRS individually as previously recommended (Goetz et al. 2008). In addition, our results showed significant improvement in the mean scores of all 3 parts in the synbiotic group 3 months after intervention compared to baseline, while in the control group, only Part II exhibited significant improvement in its mean score compared to baseline. This result comes in agreement with previous studies that reported improvement in motor symptoms, non-motor symptoms, and quality of life 8 weeks after receiving multi-strain probiotic supplementation (Ibrahim et al. 2020), as well as 12 weeks after receiving synbiotic supplementation (Mehrabani et al. 2023) compared to their baseline status. The improvement in motor symptoms in the synbiotic treated group could be explained by modification of the gut microbiota, which plays an important role in l-dopa bioavailability (Cheng et al. 2024), as well as by improving gut motility and hastening up gastric transit time, as it was proved that constipation delays gastric emptying and therefore submits l-dopa to more exposure and metabolism by peripheral dopa decarboxylase (Leta et al. 2023).

In the present study, 3 months after intervention, we noted a significant decline in TNF-α serum levels in the synbiotic group as compared to its baseline value, as well as compared to the control group. This result seems in agreement with other previous studies which reported that probiotics, prebiotics and synbiotics decreased inflammation biomarkers in individuals with various pathological conditions, including PD (Tamtaji et al. 2019; Kazemi et al. 2020). This decline in the serum level of TNF-α observed with the synbiotic group may be attributed to the ability of probiotics and inulin to down-regulate gene expression and production of pro-inflammatory cytokines (Magistrelli et al. 2019; Farabegoli et al. 2023). Furthermore, the significant positive correlation between TNF-α and Parts I, II, and III of the MDS-UPDRS revealed in our study reflects the relationship between inflammation and the symptoms of PD, as previously reported in other studies (Scalzo et al. 2010b; Lindqvist et al. 2012; Diaz et al. 2022)

During the current study, synbiotic administration for 3 months produced a significant reduction in serum MDA level in comparison to its baseline value and to the control group. Our former finding comes in accordance with other previous studies which proved the beneficial effect of probiotics and synbiotics in counteracting oxidative stress, decreasing the serum level of oxidative stress markers, and increasing the anti-oxidant biomarkers (Heshmati et al. 2018; Tamtaji et al. 2019; Bohlouli et al. 2021; Mehrabani et al. 2023). This can be explained by the ability of probiotics, as well as prebiotics to downregulate reactive oxygen species producing enzymes, such as NADPH oxidases, and their capacity to increase the level of antioxidant enzymes through activation of nuclear erythroid related factor-2 (Nrf2) (Mehrabani et al. 2023; Kavyani et al. 2024). Additionally, the significant positive correlation observed in our study between MDA and Parts I, II, and III of the MDS-UPDRS concurs with previous studies, which showed significant reduction in the UPDRS scores with anti-oxidant treatment, further implying the relationship between oxidative stress and symptoms of PD (Chang and Chen 2020).

Our findings demonstrated that, following treatment, the synbiotic group's serum levels of BDNF were significantly higher than those of the control group and their baseline value. This result seems in consonance with some previous studies which reported that probiotics increased BDNF in patients with depression and neurological conditions (Dehghani et al. 2023). The capacity of gut microbiota to produce a range of neurochemicals, including neurotransmitters and neuroactive short-chain fatty acids, specifically butyrate, which play an important role as mediators in the microbiota–gut–brain axis, subsequently increasing the production of BDNF (Molska et al. 2024) could explain the beneficial effect of synbiotic treatment in elevating BDNF levels. Furthermore, the synbiotic-treated group showed a significant negative correlation between BDNF and Parts I, II and III of the MDS-UPDRS, which reflects the role of BDNF in alleviating symptoms of PD, since BDNF was reported to maintain neuronal plasticity and adaptation to environmental challenges (Scalzo et al. 2010a; Bathina and Das 2015; Geng et al. 2023).

In the current study, there was a non-significant variation in α-Syn serum level between both study groups. To the best of our knowledge, no prior clinical study has evaluated how supplementing with probiotics or synbiotics can affect the serum level of α-Syn in humans. This non-significant difference in our result between the synbiotic and control groups can be explained by the fact that around 99% of α-Syn in human blood is carried in the RBCs, and any contamination of serum with RBCs or hemolysis could lead to inconsistency in the results of measured α-Syn in serum (Zubelzu et al. 2022).

Regarding the safety of synbiotic supplementation, our result seems in matching with the finding reported by Maherbani et al. who examined a synbiotic preparation consists of a multi-strain probiotic mixture with inulin prebiotic, and revealed that, no adverse events were reported 12 weeks after intervention (Mehrabani et al. 2023). Furthermore, in two meta-analyses, one of which analyzed 12 studies aimed at assessing the safety and efficacy of probiotics in the treatment of constipation in patients with PD and the other analyzed 11 studies aimed at assessing the effects of probiotic supplementation on PD, most of the adverse events reported were non-serious and statistically non-significant as compared to placebo, indicating good safety and tolerability of probiotics (Xie et al. 2023; Jin et al. 2024). Regarding l-dopa, it showed good tolerability and didn't provoke any l-dopa related motor complications during the 3 months treatment period, a result that comes in matching with some previous studies which reported that l-dopa induced motor complications appear after 3–5 years of continuous l-dopa use (Salat and Tolosa 2013; Pandey and Srivanitchapoom 2017). Moreover, neither l-dopa induced psychiatric adverse effects nor l-dopa induced dopamine dysregulation syndrome (DDS) were observed during our study, which seems in agreement with previous reports that demonstrated that, l-dopa related psychiatric complications appear in less than 5% of patients who started l-dopa therapy for the first time, and that DDS only appear after long period of l-dopa administration (Plewnia et al. 2024).

Our study's notable points of strengths include its design as a randomized controlled parallel study and, to the best of our knowledge, being the first study to compare the mean change from baseline scores of each individual part of the MDS-UPDRS after 3 months of treatment between both study groups, as well as to examine both the clinical and biochemical effects of a synbiotic preparation containing a single probiotic strain, Lactobacillus acidophilus, with inulin prebiotic, and emphasizing the correlation between the clinical result with the biological markers implicated in the pathophysiology of PD. Our study does, however, have some limitations, such as its open-label design, not being a placebo controlled double blind study, short follow-up duration, and comparatively small sample size. Thus, additional large-scale, more longitudinal research and the inclusion of a placebo group are further required to validate the encouraging findings of this study as well as to assess the impact of the synbiotic supplementation on the MDS-UPDRS part IV score (l-dopa induced motor complications) after longer duration of therapy.

## Conclusion

The current study's findings showed that administering synbiotics, particularly Lactobacillus acidophilus with inulin, as an adjuvant with l-dopa in PD patients appears to be safe and effective, and represent a promising neuro-protective and therapeutic agent in treatment of PD, since it increased the levels of the neuroprotective neurotrophin BDNF, and decreased the levels of the oxidative stress marker MDA, and the inflammatory marker TNF-α, which was linked to a clinical improvement in both motor and non-motor symptoms compared to control.

## Data Availability

Data are available upon reasonable request from the corresponding author.
